# Family Socioeconomic Status at Birth and Youth Impulsivity at Age 15; Blacks’ Diminished Return

**DOI:** 10.3390/children5050058

**Published:** 2018-05-01

**Authors:** Shervin Assari, Cleopatra Howard Caldwell, Ron Mincy

**Affiliations:** 1 Center for Research on Ethnicity, Culture and Health, School of Public Health, University of Michigan, Ann Arbor, MI 48109, USA; cleoc@umich.edu; 2 Department of Psychiatry, University of Michigan, Ann Arbor, MI 48109, USA; 3 Department of Health Behavior & Health Education, University of Michigan School of Public Health, Ann Arbor, MI 48109, USA; 4 Columbia School of Social Work, Columbia University, New York, NY 10027, USA; rmincysr@gmail.com

**Keywords:** race, ethnicity, social class, education, socioeconomic status, income, social determinants of health, impulsivity

## Abstract

Minorities’ Diminished Return theory suggests that health effects of socioeconomic status (SES) are systemically smaller for racial and ethnic minorities compared to Whites. To test the relevance of Minorities’ Diminished Return theory for youth impulsivity, we investigated Black–White differences in the effects of family SES at birth on subsequent youth impulsivity at age 15. Data came from the Fragile Families and Child Wellbeing Study (FFCWS), 1998–2016, a 15-year longitudinal study of urban families from the birth of their children to age 15. This analysis included 1931 families who were either White (*n* = 495) or Black (*n* = 1436). The independent variables of this study were family income, maternal education, and family structure at birth. Youth impulsivity at age 15 was the dependent variable. Gender was the covariate and race was the focal moderator. We ran linear regressions in the overall sample and specific to each race. In the overall sample, higher household income (b = −0.01, 95% CI = −0.01 to 0.00) and maternal education (b = −0.24, 95% CI = −0.44 to −0.04) at birth were associated with lower youth impulsivity at age 15, independent of race, gender, and family structure. A significant interaction was found between race and household income at birth (b = 0.01, 95% CI = 0.00 to 0.02) on subsequent youth impulsivity, which was indicative of a stronger protective effect for Whites compared to Blacks. Blacks’ diminished return exists for the long-term protective effects of family income at birth against subsequent youth impulsivity. The relative disadvantage of Blacks in comparison to Whites is in line with a growing literature showing that Black families gain less from high SES, which is possibly due to the existing structural racism in the US.

## 1. Background

Although the protective effects of socioeconomic status (SES) on a wide range of health outcomes are well established [1,2,3], these gains are unequal across demographic groups. According to the Minorities’ Diminished Return theory [4,5,6], racial and ethnic minorities constantly gain less tangible outcomes from the same SES indicators compared to Whites [4]. Minorities’ Diminished Return theory argues that race/ethnicity and SES are two different and overlapping axes of social stratification, that both collectively, and interactively explain racial disparities in health and health behaviors [5,6]. Although most of the research on Minorities’ Diminished Return theory is on physical and mental health [7,8], some recent studies suggest that it also impacts risk behaviors such as substance use and suicide [4,9,10].

Minorities’ Diminished Return theory is supported by extensive empirical evidence [5,6,11]. Among adults, high SES (e.g., education and income) better protects Whites than Blacks against drinking [9], depression [7], suicide [10], chronic disease [7], and body mass index (BMI) [8]. Similar results are also shown for adolescents [8]. For instance, in a longitudinal study, race altered the protective effect of family SES at birth on youth BMI at age 15, with protective effects present for White but not Black youth [8]. In another recent national study, the effects of parental education on families’ ability to escape poverty was larger for Whites compared to Blacks [12].

Diminished returns among Blacks have been attributed to a wide range of underlying mechanisms, with racism and discrimination being among the most intuitive explanations. Due to the existing structural racism, significant qualitative differences exist in the daily lives of White and Black families. Such differences in life conditions reduce Black families’ abilities to actualize their potentials and to gain tangible outcomes from their SES resources. As in the US, several institutions treat the majority and minorities differently, Blacks maintain lower access to the opportunity structure than Whites. Regardless of their SES, Blacks face discrimination in the labor market, education, and correctional setting. As a result, each SES indicator result in smaller changes in the daily lives of Blacks than Whites [13,14]. Education generates better occupation and more income, income generates more purchasing power and wealth, and employment generates better life conditions for Whites compared to Blacks [7,9,10,15,16,17,18]. The history of slavery combined with facing disproportionately higher rates of societal barriers on a daily basis hinder Blacks from gaining the most possible benefits from a new resource that becomes available to them [13,19,20].

Impulsivity, which can be defined as “a predisposition towards rapid, unplanned reactions to internal or external stimuli without regard to the negative consequences of these reactions to the impulsive individual or to others,” is a major risk factor for a wide range of high risk behaviors [21] (p. 1784). Youth with high impulsivity show poor inhibitory control, rapid response, and inability to delay gratification [22,23]. Impulsivity is higher in Blacks than Whites [24]. Low SES is also associated with higher impulsivity [25]. Impulsivity is linked to drug use, delinquent behaviors, and suicidality [21].

Low levels of impulsivity and high emotion regulation are two mechanisms by which high SES protects populations against high risk behaviors [26,27]. Education helps populations gain better impulse-control abilities and not operate on their urges and impulses [28]. Better emotion regulation and lower impulsivity are main reasons why high SES youth are at lower risks of smoking, drug use, and suicide [29]. However, because SES has smaller effects on risk behaviors for Black than White adults [9,10], SES may have smaller effects on impulse control for Black than White youth. We are, however, unaware of any previous studies on Black–White differences in the effects of family SES on youth impulsivity over time.

Research on affective neuroscience and developmental psychology [30] has shown that racial minority status [24,31,32] and SES such as living in poverty [33,34] and low parental education [35,36] are risk factors for poor impulse control and emotion regulation for youth. Extensive research has linked poor impulse control as a robust risk factors for children’s and adults’ negative outcomes such as delinquency, substance abuse, and suicide [25,37,38,39]. That is, impulse control (defined as inhibiting an automatic response in order to successfully complete a goal) has a central role in determining disparities in problem behaviors such as substance use [40]. Low-income increases behavioral disinhibition, while high SES youth are better able to regulate and control their emotions and behaviors [40]. Disinhibition and impulsivity predict risky behaviors such as alcohol use in youth and adults [41,42,43,44,45,46,47].

To extend the existing knowledge on the Minorities’ Diminished Return theory, this study used data from the Fragile Families and Child Wellbeing Study (FFCWS) to explore Black–White differences in the effects of family SES (household income and maternal education) at birth on youth impulsivity at age 15.

## 2. Materials and Methods

### 2.1. Design and Setting

Fragile Families and Child Wellbeing Study (FFCWS), 1998–2016, is an ongoing longitudinal study of economically fragile families. Details on sampling and methods of the FFCWS are available elsewhere [48]. Here, we briefly describe the FFCWS methods.

### 2.2. FFCWS Sample and Sampling

FFCWS enrolled a random sample of urban families from 20 US cities with a population of 200,000 people or more. The FFCWS has oversampled non-married and Black and Hispanic couples [48]. Most of the FFCWS participants were non-marital, low SES, minority families. As a result, this national sample is not representative of the US general population. The FFCWS original sample composed of 4655 families (2407 Blacks, 1354 Hispanics, and 894 Whites). 

### 2.3. Analytical Sample in the Current Study

Data for this analysis came from the first and sixth waves of the FFCWS. This analysis was limited to Black and White families that had data on family SES at baseline (wave 1) and youth impulsivity 15 years later (wave 6). The analytical sample was composed of 1931 families who were either White (*n* = 495) or Black (*n* = 1436) families.

### 2.4. Study Variables

#### 2.4.1. Independent Variables

The main family SES indicators included household income, maternal education, and family structure at birth. All these SES indicators were measured at the wave 1. Maternal education was treated as a continuous variable: (1) less than high school, (2) high school completed, (3) some college education, and (4) college completed/graduate studies. Household income was also treated as a continuous measure, with higher score indicating higher SES. Family structure was treated as a dichotomous variable, based on the marital status reported by the mother at wave 1. This variable was operationalized as married families 1 versus non-married families 0.

#### 2.4.2. Covariate

Gender was the only covariate in the study. Gender was operationalized as a dichotomous variable, with males (reference category) and females.

#### 2.4.3. Main Dependent Variables

Based on a measure developed by Dickman in 1990 [49], the following items were used to measure impulsivity. (1). I don’t spend enough time thinking over a situation before I act, (2) I often say and do things without considering the consequences, (3) I often make up my mind without taking the time to consider the situation from all angles, (4) I often say whatever comes into my head without thinking first, and (5) I often get into trouble because I don’t think before I act. Response items included (1) Not true, (2) Sometimes true, and (3) Often true. We calculated a sum score, which ranged from 5 to 15, with a high score indicating higher impulsivity (alpha = 0.664). These items are widely used to measure impulsivity [49,50,51].

#### 2.4.4. Moderator

Race was the focal moderator. Race was operationalized as a dichotomous variable, with Whites (reference category) and Blacks.

### 2.5. Statistical Analysis

Data analysis was done using the SPSS 22.0 (SPSS Inc., Chicago, IL, USA). For univariate analysis, frequency (%) and mean (standard deviation) were reported. For bivariate analysis, Pearson correlation test was used in the overall sample and separately for Whites and Blacks. For multi-variable analysis, we used linear regression models in the overall sample and also specific to race. 

In all linear regression models, youth impulsivity at age 15 was the dependent variable, household income, maternal education, and family structure at birth were the independent variables, and gender was the covariate. *Model 1* and *Model 2* were conducted in the overall sample. *Model 1* only included the main effects of SES indicators and covariates. *Model 2*, also included three race by SES interaction terms [(1) household income × race, (2) maternal education × race, and (3) family structure × race]. *Model 3* and *Model 4* tested the effects of SES indicators in White and Black families, respectively. Unstandardized adjusted regression coefficients (b) and their 95% confidence intervals (CIs) were reported. *p* values less than 0.05 were considered significant.

### 2.6. Ethical Considerations

The Princeton University Institutional Review Board (IRB) approved the FFCWS study protocol. Youth legal guardians/parents provided informed consent. Youth provided assent. Data were gathered, kept, and analyzed anonymously. Respondents received financial compensation.

## 3. Results

### 3.1. Descriptive Statistics

This study included 1931 families who were either White (*n* = 495) or Black (*n* = 1436). These families were followed from birth of their child to the age of 15. 

Table 1 provides descriptive statistics of the study variables for the overall sample, as well as for each race. Household income was higher for White compared to Black families. Most White families were married couples, while most Black families were of unmarried couples. Maternal education at birth was higher for White compared to Black families. Finally, youth impulsivity at age 15 was higher in Blacks than Whites.

### 3.2. Bivariate Correlations

Table 2 summarizes the results of three sets of bivariate correlations. The first set is for the overall sample, the second one is for Whites and the last set is for Blacks. Family SES at birth was negatively associated with youth impulsivity at age 15 in the overall sample. These associations were present for Whites and Blacks.

### 3.3. Family SES at Birth and Youth Impulsivity 15 Years Later

Table 3 summarizes the results of two linear regression models in the overall sample to test the association between family SES (household income, maternal education, and family structure) at birth and youth impulsivity at age 15. *Model 1*, which did not include any interaction terms, showed that high household income (b = −0.01, 95% CI = −0.01 to 0.00) and maternal education (b = −0.24, 95% CI = −0.44 to −0.04) at birth were associated with lower impulsivity of youth at age 15 in the overall sample. *Model 2*, which included three interaction terms between race and SES indicators (maternal education, family structure, and household income), showed a significant interaction between race and household income (b = 0.01, 95% CI = 0.00 to 0.02) on youth impulsivity at age 15, suggesting larger gains for Whites than Blacks.

Table 4 summarizes the results of two linear regression models by race. *Model 3* and *Model 4*, which were stratified regressions, showed an association between family income at birth and impulsivity at age 15 for White but not Black youth. There was also a protective association between maternal education and impulsivity for Whites (b = −0.01, 95% CI = −0.02 to 0.00) but not for Blacks (b = 0.00, 95% CI = −0.01 to 0.01). There was also an association between family structure and impulsivity for Blacks (b = −0.67, 95% CI= −1.30 to −0.04) but not for Whites.

## 4. Discussion

The current study explored the Black–White differences in the effects of three family SES indicators at birth on subsequent impulsivity of youth at age 15. The results were suggestive that Minorities’ Diminished Return theory holds for the long-term effects of household income on youth impulsivity. We did not, however, find any interactions between race and maternal education or family structure at birth on subsequent youth impulsivity 15 years later.

Our findings are consistent with the Minorities’ Diminished Return theory [5,6,8], defined as systemically smaller health effects of very same SES indicators for Black than White families [10,13,16]. Effects of individual level education and income on drinking [9], BMI, insomnia, physical activity [9], depression [7], suicide [10], and mortality [16] are found to be smaller for Blacks than Whites. Not only does SES generate less health benefits and wellbeing for Blacks than Whites [5,6], but high SES sometimes operates as a risk factor for poor mental health of Blacks [7,10,11,19,20,52,53,54,55,56,57,58]. For instance, high education and income may increase the risk of depression for Blacks, particularly Black men and boys [54,57]. This is potentially because discrimination increases as SES increases for Black males [52,56,58].

Blacks’ Diminished Return does not attribute the observed Black-White differences to Blacks’ lower innate ability to turn their resources to tangible outcomes. Blacks’ smaller gains, instead of Blacks’ culture or biology, is a function of history and systemic racism in the US. Decades after slavery ended and decades after the civil right movement, racism is still a core element of the US social structure. Racism holds Black families behind at multiple levels, and constantly reduces their power to leverage the system and mobilize their economic resources and psychological assets [48,49,50,51]. Until the day when race and skin color no longer shape sub-populations access to the opportunity structure, and only when all groups are equally treated by society, a true equality and social justice is not achievable. Without such a change, US system will continue to fail Black families.

Black families pay extra costs for their upward social mobility [11,55,59,60], which minimizes their gains of high SES [19,56,57,58]. The US political and economic system has historically been, and continues to be, designed to maintain the economic advantage of Whites. Maximizing gains for the White majority groups sometimes comes with an unintended cost of sub-optimal gains for other social groups. Blacks who have high aspirations may attain a high level education, however, this education does not result in an equally high pay off by means of high paying jobs, purchasing power, wealth, life conditions, and health as compared to Whites [5,6].

The current findings are also supported by other theoretical models. Other than Blacks’ Diminished Return [5,6,61,62,63], the results are in line with the Link and Phelan’s (1995) Fundamental Cause Theory, which conceptualizes low SES as a fundamental and root cause for a wide range of health problems [1,2,3]. The results are also in line with Bronfenbrenner’s Ecological Model of Human Development [22] and Family Financial Stress Model [64,65,66], which conceptualize family SES as a major context for positive development. Finally, the results support the Life Course Developmental Approach, which suggests early exposures have long-term consequences that are detectable decades later [67,68,69,70].

Although in bivariate analysis, race was found to be associated with impulsivity, this association did not remain in the multivariable models that controlled for SES. This finding is supported by the studies showing higher impulsivity in Blacks [24] and low SES individuals [25] compared to Whites and high SES individuals. Previous research has also shown that after controlling for SES, the association between race and high risk behaviors disappear, which indicates it is not race but SES that explains high impulsivity among Blacks [59].

This study conceptualized impulsivity as a risk rather than protective factors. There are, however, other approaches to conceptualization of impulsivity [71,72]. For instance, Dickman in 1990 argued that impulsivity can be functional and dysfunctional [49], which was not distanced in thus study. We defined impulsivity as “a predisposition towards rapid, unplanned reactions to internal or external stimuli without regard to the negative consequences of these reactions to the impulsive individual or to others”, which is constantly shown as a major risk factor for a wide range of high risk behaviors [21] (p. 1784). This view has extensive empirical support [39,40,41,42,43,44,45,46,47].

### 4.1. Public Policy Implications

SES does not generate the same tangible results for all racial groups, with high SES placing Blacks and other minorities at a systemic disadvantage relative to the majority group. As it was mentioned before [5,6], policy solutions should go beyond equalizing access. In addition to aiming for reducing inequalities in SES across groups, policies should address societal and structural barriers in the lives of minority groups. It should be ensured that all groups receive the same quality of education, which requires investment on inner city schools. Educational policies should protect minority students against discrimination at school. At the same time, Black families may require some extra help to leverage their available SES resources such as education and income. Public and economic policies should aim to reduce the inequalities in the degree by which education generates employment, income, and insurance for racial groups. Inequalities in the effects are among neglected causes of racial health disparities. Other than comparison of averages, researchers should compare social groups for the magnitude of the gain that follows each protective factor.

### 4.2. Public Health Implications

Our results on differential effects of family income on impulse control is particularly important given the central role of low SES and impulsivity as risk factors for a wide range of health risk behaviors such as smoking, drug use, suicide, violence, and early sexual debut [73]. Traditionally, research has documented a lower impulse control among low SES and minority individuals [24]. Our study suggests that the effects of race, SES, and impulse control are multiplicative and non-linear. So, the gain of SES in reducing impulsivity is not equal across racial groups, thus SES and impulse control may be differently salient for risk taking behaviors among diverse racial groups with similar SES levels [74]. Our findings suggest that unequal gains from SES should be seen as another reason why Black youth show lower impulse control than Whites [24,25]. As the effect of SES on enhancing impulse control abilities is diminished for Black families, Black youth from high SES families are still at a risk to operate on their urges and impulses, which is disproportionate to their SES [28]. These findings may have implications for public health programs and clinicians who wish to enhance emotion regulation and reduce impulsivity as strategy to prevent risk behaviors such as substance use, sexual debut, aggressive behaviors, and suicide [29]. 

### 4.3. Limitations and Future Research

Several limitations are worthy of notation. First, family SES was conceptualized as fixed, while it changes over time. Future research should also examine SES as a time varying covariate. SES is hard to pinpoint, and small measurement errors impact disparities conclusions, and risk of residual confounding due to measurement error exists [75]. Hence models with latent variables instead might be more sensitive. Our SES indicators were not comprehensive. SES indicators such as household size, employment, occupation type, sources of income, and wealth were not explored. Other SES measures such as various sources of income, receiving social and public programs such as food stamps, and housing and food insecurity should be investigated. Various SES predictors may be themselves causally related, for instance maternal education impacts current and future income, so future models should allow various links between SES measures. The study included maternal education, and father presence and paternal education and employment were left out. These variables should be covered in future research. SES indicators that are not limited to individual level should be also included in future research. Availability of resources, racial composition, and other contextual factors in family, community, and schools should not be ignored. Parental and child physical and mental health should be studied. Future research should not limit itself to racial differences and should test intersections of a wide range of identities such as ethnicity, gender, immigration status, nativity, place, and SES. Cronbach’s alpha for the dependent variable was low, which suggests the results should be replicated in other samples.

Another major limitation was that family types are not comparable for Blacks and Whites in the FFCWS. While most Black children are born to non-married parents, most White children are born to married parents. Although we controlled for family structure, controlling for marital status may not fully solve this problem. Among Blacks, marital status and household income are so highly correlated, that after controlling for marital status, there is no independent contribution of household income to impulsivity. This is partially because only 12% of the children in Black families are born to married couples, and these “outlier” families are composed of the highest earning mothers and fathers in the Black subsample. The economic situation and family processes of these married Black families are very different from the rest of the sample, and may require more research. On the other hand, 60% of the white subsample is married, therefore there is a greater variation in the household income among married and non-married White families than among the married Black families. For these reasons, more research is needed on the effects of marital status, income and education in White and Black families on processes that reduce youth impulsivity.

Despite above limitations, this study still extends the existing knowledge on Blacks Diminished Return by showing for the first time that household income better reduces impulsivity of White youth than Black youth. Although we did not have multiple observations of our outcome, the study used a longitudinal dataset and had 15 years of follow up. Thus, despite lack of repeated measurements, the study was unique in large sample size and long follow up. Other strength of the current study was national sample.

In response to the above limitations, there is a need to explore multi-level causes of Blacks Diminished Return. Future research should test how changes in the family SES generate health outcomes for social groups. It is still unclear what the underlying mechanisms that result in Black-White differences in the gain from SES may be. Causes may be multi-level, as structural factors, discrimination at multi-levels, parental behaviors (communication and time spent on the child), school and quality of school and teachers, availability of educational resources, and child behaviors (motivation, dedication, self-efficacy, time spent on homework) may have some roles. There is a need to investigate the economic, family, and welfare policies and programs that can potentially minimize the diminished returns of SES indicators among Blacks [6,8,56]. Future research may also study how variation in economic and welfare policies across states contribute to these inequalities by race. 

## 5. Conclusions

In summary, the Black–White differences observed here are in line with the Minorities’ Diminished Return theory. Racial inequalities in the magnitude of the gains from SES are unacceptable and should be minimized. Policies should help Black families to gain as much as Whites from their resources. Policy solutions should be multi-level, should go beyond access, and should address structural barriers that disproportionately affect Blacks and other racial minority groups.

## Figures and Tables

**Table 1:** Descriptive statistics in the overall sample and by race (*n* = 1931).

Characteristics	All *n* = 1931		Whites *n* = 495		Blacks *n* = 1436	
	*n*	%	*n*	%	*n*	%
**Family Race**						
White	495	25.60	495	100.00	-	-
Black	1436	74.40	-	-	1436	100%
**Child Gender**						
Male	981	50.83	-	-	-	-
Female	950	49.17	-	-	-	-
Family Structure (Married Parents) at Baseline *^,a^						
No	1462	75.70	198	40.00	1264	88.03
Yes	469	24.30	297	60.00	172	11.97
	**Mean**	**SD**	**Mean**	**SD**	**Mean**	**SD**
Household Income (USD1000) at Baseline *^,b^	33.80	33.16	61.21	41.74	24.35	23.03
Maternal Education at Baseline *^,b^	2.25	1.00	2.90	1.05	2.02	0.88
Youth Impulsivity at Age 15 *^,b^	7.40	3.68	6.60	3.62	7.67	3.66

* *p* < 0.05 for comparison of Blacks and Whites; ^a^ Pearson Chi square test; ^b^ Independent sample *t* test.

**Table 2:** Bivariate correlations in the overall sample and across races (*n* = 1931).

Characteristics	1	2	3	4	5	6
**Overall sample (*n* = 1931)**						
1 Race (Black)	1.00	0.01	−0.49 **	−0.38 **	−0.49 **	0.13 **
2 Gender (Female)		1.00	−0.00	−0.02	0.02	−0.02
3 Family Structure (Married Parents) at Baseline			1.00	0.50 **	0.56 **	−0.14 **
4 Maternal Education at Baseline				1.00	0.55 **	−0.14 **
5 Household Income (USD1000) at Baseline					1.00	−0.16 **
6 Youth Impulsivity at age 15						1
**Whites (*n* = 495)**						
2 Gender (Female)		1.00	−0.05	−0.04	−0.01	−0.11 *
3 Family Structure (Married Parents) at Baseline			1.00	0.56 **	0.50 **	−0.10 *
4 Maternal Education at Baseline				1.00	0.52 **	−0.17 **
5 Household Income (USD1000) at Baseline					1.00	−0.20 **
6 Youth Impulsivity at age 15						1.00
**Blacks (*n* = 1436)**						
2 Gender (Female)		1.00	0.03	−0.01	0.06 *	0.01
3 Family Structure (Married Parents) at Baseline			1.00	0.29 **	0.35 **	−0.08 **
4 Maternal Education at Baseline				1.00	0.43 **	−0.08 **
5 Household Income at Baseline					1.00	−0.06 *
6 Youth Impulsivity at age 15						1.00

Pearson Correlations were used; * *p* < 0.05; ** *p* < 0.01.

**Table 3:** Summary of linear regression models in the overall sample (*n* = 1931).

Characteristics	B	95%CI	B	95%CI
	*Model 1*		*Model 2*	
Race (Black)	0.40	−0.05–0.85	−0.09	−1.14–0.97
Gender (Female)	−0.16	−0.48–0.16	−0.16	−0.49–0.16
Family Structure (Married Parents) at Baseline	−0.33	−0.82–0.16	0.30	−0.52–1.12
Maternal Education at Baseline	−0.24 *	−0.44–−0.04	−0.38	−0.76–0.01
Household Income (USD1000) at Baseline	−0.01 *	−0.01–0.00	−0.01 **	−0.02–0.00
Race × Married			−0.96	−1.99–0.07
Race × Maternal Education			0.15	−0.30–0.61
Race × Household Income (USD1000)			0.01 *	0.00–0.02
Intercept	8.08 ***	7.44–8.71	8.44 ***	7.48–9.40

Overall models are statistically significant; Outcome: Youth Impulsivity at Age 15; Confidence Interval (CI); * *p* < 0.05; ** *p* < 0.01; *** *p* < 0.001.

**Table 4:** Summary of linear regression models across races.

Characteristics	B	95%CI	B	95%CI
	Whites *n* = 495		Blacks *n* = 1436	
	*Model 3*		*Model 4*	
Gender (Female)	−0.85 **	−1.47–−0.23	0.07	−0.31–0.45
Family Structure (Married Parents) at Baseline	0.27	−0.53–1.07	−0.67 **	−1.30–−0.04
Maternal Education at Baseline	−0.39 *	−0.76–−0.01	−0.22	−0.46–0.02
Household Income (USD1000) at Baseline	−0.01 **	−0.02–0.00	0.00	−0.01–0.01
Intercept	8.80 ***	7.83–9.78	8.23 ***	7.71–8.74

Overall models are statistically significant; Outcome: Youth Impulsivity at Age 15; Confidence Interval (CI); * *p* < 0.05; ** *p* < 0.01; *** *p* < 0.001.
